# Transcriptional Analyses of Genes Related to Fodder Qualities in Giant Leucaena Under Different Stress Environments

**DOI:** 10.3389/fpls.2022.885366

**Published:** 2022-06-16

**Authors:** Ahmed M. Bageel, Aaron Kam, Dulal Borthakur

**Affiliations:** Department of Molecular Biosciences and Bioengineering, University of Hawai’i at Mānoa, Honolulu, HI, United States

**Keywords:** giant leucaena, mimosine, tannin, environmental stress, gene expression, fodder quality

## Abstract

*Leucaena leucocephala* subsp. *glabrata* (giant leucaena) is a tree legume, whose foliage is used as a fodder for animals because of its high protein content. In spite of being a highly nutritious fodder, giant leucaena foliage has two undesirable secondary metabolites, mimosine and tannin. The amounts of mimosine and tannin in giant leucaena foliage are known to vary under different environmental conditions. Giant leucaena was grown under different salinity, pH and nitrogen availability conditions. It produced the highest amounts of mimosine at pH 6.0–7.0, whereas, variation in soil pH did not affect tannin concentrations. Salinity stress had negative effects on both mimosine and tannin concentrations, while nitrogen abundance promoted both mimosine and tannin production. Seven genes for mimosine and tannin metabolism were isolated from a transcriptome library of giant leucaena. These were mimosine synthase, mimosinase, chalcone synthase, flavanone 3β-hydroxylase, dihydroflavonol reductase, leucoanthocyanidin reductase, and anthocyanidin reductase. The highest level of mimosine synthase activity was observed in the absence of salt in the soils. Mimosine synthase activities had strong positive correlation with mimosine concentrations in the foliage (R^2^ = 0.78) whereas mimosinase expression did not appear to have a direct relationship with salt concentrations. The expression of mimosine synthase was significantly higher in the leucaena foliage under nitrogen abundant condition than in nitrogen deficiency conditions, while mimosinase expression was significantly higher under nitrogen deficiency condition than in nitrogen abundance conditions. Mimosine concentrations in the foliage were positively correlated with the expression levels of mimosine synthase but not mimosinase. Similarly, the concentrations of tannin were positively correlated with expression levels of dihydroflavonol reductase, leucoanthocyanidin reductase, and anthocyanidin reductase. Understanding of the environmental conditions that promote or inhibit transcription of the genes for mimosine and tannin biosynthesis should help to design environmental conditions that inhibit transcription of these genes, resulting in reduced levels of these compounds in the leucaena foliage.

## Introduction

Giant leucaena is a tree legume, which is widely grown in many tropical and subtropical countries as a source for nutritious fodder. It can be managed as a dwarf bush by repeated harvest of its foliage by pruning (Ishihara et al., 2018). On the other hand, common leucaena grows as a bushy shrub even without pruning. Giant leucaena is ∼2.5 times more productive than common leucaena for fodder production (Bageel et al., 2020). Because of the high protein content (18–30%) in its foliage, it is often described as the “alfalfa of the tropics” (Bageel et al., 2020). Although leucaena is considered to be a nutritious fodder, it has two major secondary metabolites, mimosine and tannin, which reduce the fodder quality if they are present in high concentrations. These two metabolites vary in their concentrations depending on the environmental conditions that leucaena is exposed to (Bageel and Borthakur, 2022).

Mimosine is a non-protein amino acid present in all parts of the leucaena plant; young leaves, growing shoot tips, and seeds contain the highest concentration of mimosine (Xuan et al., 2006). The concentrations of mimosine in the foliage and other parts of the leucaena plants change in response to various environmental stresses; therefore, mimosine has been described as a stress-response molecule (Honda and Borthakur, 2021). Mimosine is also secreted in the root exudates to the rhizosphere, where it works as a phytosiderophore and facilitates metallic cation uptake from the soil (Honda and Borthakur, 2020). It also serves as an antioxidant that mitigates free radicals and limits oxidative damage (Honda and Borthakur, 2021). On the other hand, mimosine is considered a toxic compound that negatively affects animals when ingested in high amounts. According to Szyszka and Ter Meulen (1984), consuming low amounts of mimosine (<0.14 g/kg BW) does not cause harmful effects in animals. Therefore, it is desirable to reduce mimosine concentrations in leucaena through inter-varietal and inter-species crosses, and selection. Recently, two key genes that are responsible for synthesis and breakdown of mimosine have been identified; they are mimosine synthase and mimosinase, respectively (Negi et al., 2014; Harun-Ur-Rashid et al., 2018).

Tannins are phenolic compounds that play important roles in forage quality. High amounts of tannins have negative impacts on the foliage quality, it reduces ruminants’ digestion (Reed, 1995). On the other hand, moderate amounts of tannins have positive impacts on foliage quality by disrupting protein foam in the rumen that prevents rumen pasture bloating (McMahon et al., 2000). In addition, moderate amounts of tannins reduce the number of internal parasites and increase the titer of small peptides and amino acids without compromising total digestion (Niezen et al., 1995). As a result, livestock can more efficiently produce meat, fur, and milk by consuming foliage with moderate concentrations of tannins compared with tannin-free foliage (Min et al., 1998).

**FIGURE 1:**
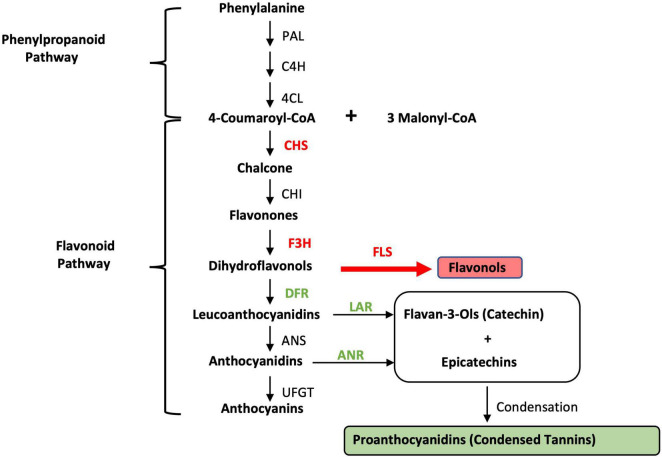
Condensed tannin biosynthesis pathway. PAL, phenylalanine ammonia-lyase; C4H, cinnamic acid 4-hydroxylase; 4CL, 4-coumarate; CHS, chalcone synthase; CHI, chalcone isomerase; F3H, flavanone 3β-hydroxylase; FLS, flavonol synthase; DFR, dihydroflavonol reductase; LAR, leucoanthocyanidin reductase; ANS, anthocyanidin synthase; ANR, anthocyanidin reductase; UFGT, UDP-glucose flavonoid 3-*O* glucosyltransferase. The genes/enzymes used in this study are shown in bold.

Giant leucaena foliage is considered superior to common leucaena foliage for a number of reasons: (i) it remains in juvenile stage for a relatively longer period of time, making it more palatable and digestible; (ii) it produces much higher amounts of foliage; (iii) it produces much less pods and seeds; and (iv) it produces less amounts of tannins. Cultivars such as K636 and KX2 are known to have low amounts of tannins (Bageel et al., 2020). It was shown previously that foliage yield, and the concentrations of protein, mimosine and tannin change under different soil salinity and pH conditions. The goal of the present study was to determine the expression of a number of genes related to fodder quality under different salinity, pH conditions, and nitrogen availability. The leucaena genome has not been sequenced and genes for tannin biosynthesis have not been isolated from leucaena. Therefore, the coding sequences for these genes were isolated through transcriptome sequencing. To determine if the coding sequences for these genes in giant leucaena differ from those of common leucaena, these sequences were isolated from the transcriptome libraries of both giant and common leucaena and compared. For studying expression of genes related to fodder quality, seven genes were selected; two for mimosine metabolism (mimosine synthase and mimosinase), and five genes for tannin biosynthesis. Among these five genes, two genes were for general phenylpropanoid pathway and three genes were specific for tannin biosynthesis. The phenylpropanoid pathway genes were chalcone synthase (CHS), and flavanone 3β-hydroxylase (F3H) and genes for tannin biosynthesis were dihydroflavonol reductase (DFR), leucoanthocyanidin reductase (LAR), and anthocyanidin reductase (ANR) (Figure 1).

## Materials and Methods

### Transcriptome Analysis

#### Plant Material

Seeds of giant (K636) and common leucaena were collected from Waimanalo Research Station, University of Hawaii. The seeds were scarified, germinated, and grown in pots as described earlier (Bageel and Borthakur, 2022). Three-months old seedlings (shoots and roots) were harvested, rinsed with water, and placed immediately in four different containers (giant shoots, giant roots, common shoots, and common roots) filled with liquid nitrogen.

#### RNA Extraction

Total RNA was extracted and purified using the reagents and protocol described by Ishihara et al. (2016). To remove contamination of phenolic compounds, the isolated total RNA samples were precipitated with glycogen and resuspended in RNAse-free dH_2_O. To confirm the RNA’s quality, the RNA yields were analyzed by checking the RNA Integrity Numbers (RIN) using Agilent Technologies 2100 Bioanalyzer, and RIN were 7.30, indicating acceptable level yield and purity (Supplementary Figure 1). The samples were then treated with TURBO DNA free Kit to remove possible DNA contamination according to manufacturer’s instructions.

#### Poly-A RNA Sequencing

Cleaned total RNA extracted from the foliage and root samples were combined in two Eppendorf tubes, one for giant leucaena and the other for common leucaena. These two sample tubes were sent to the LC Sciences company for transcriptome sequencing where Illumina’s TruSeq-stranded-mRNA sample preparation protocol was followed. LC Sciences purified mRNA from the samples by using oligo-(dT) magnetic beads which attach to poly(A)-tails of mRNAs. All mRNA were fragmented to small contigs by using divalent cation buffer in high temperature. Paired-end sequencing on the resulting fragments was performed using Illumina’s NovaSeq 6000 sequencing system which produced the raw reads.

#### Transcriptome Assembly

The raw reads were cleaned using Cutadapt-3.4 to remove bases with a quality score less than 20, alongside contamination from the Illumina universal adapters used during sequencing. The removal of adapter contaminations and low-quality reads were confirmed by using FastQC-0.11.9 resulting in high quality reads. The high-quality reads were then *de novo* assembled into a transcriptome using Trinity-2.12.0 that contains predicted mRNA transcripts. Transcripts generated by Trinity were clustered into groups based on shared component sequences that are loosely considered to represent the same gene. The longest transcript in each of the gene clusters was designated as a unigene and was used as a representative for downstream gene-level analyses.

#### Functional Annotation

The primary unigene library was annotated by the Non-Redundant (nr), Swissprot, Gene Ontology (GO), and Kyoto Encyclopedia of Genes and Genomes (KEGG) databases. The translated amino acid sequences for each unigene were compared against entries in the databases using DIAMOND accelerated BLASTx and alignments with an e-value < 1e^–5^ were used as annotations.

However, these annotations are limited to the unigene cluster as a whole and do not provide the specific, transcript-level information that is needed to create qRT-PCR primers. To determine the appropriate coding nucleotide sequences from the transcripts, annotations from each database were searched for labels of the desired genes. Because names can vary between databases and entries, annotations containing aliases, alternate names, and protein isoforms of our desired genes were also considered. Unigenes with annotations of interest had their related transcripts extracted and compared to the nr and Swissprot databases using blastx. Transcripts were selected if either of their translated amino acid alignments covered a large portion of a desired protein in its alignment; had a high (>90%) similarity; and contained the protein’s functionally important domains and residues. These transcripts had their coding nucleotides for the aligned areas used as representative sequences for creating qRT-PCR primers.

### Biochemical Assays and Gene Expression

#### Plant Material

Approximately 100 giant leucaena seedlings were grown in the greenhouse in large pots containing potting mix planting soil (Sam’s Club) for 1 year, and only irrigated with tap water. Thus, the planting soil has lost most of its minerals, and that was confirmed by the EC probe where the dripped water from the soil pots showed close mineral concentration that was shown on sink water. These seedlings were used in the following three experiments.

(i) Twenty of the seedlings, which showed uniformity in size and health condition, were selected for the experiment. These plants were pruned to develop fresh foliage uniformly, and grouped into four groups representing four different salinity levels (Zero, 50, 100, and 150 mM NaCl). Every group was irrigated every other day with 1/18 strength of Hoagland solution adjusted to pH 7.0 and the salinity level, which the group is representing for 3 months. Then, 300 mg of young leaves were harvested and placed in liquid nitrogen immediately, and kept in −80°C for RNA extraction. The rest of the green foliage was harvested for mimosine and tannin quantifications.
(ii) Another group of twenty-five seedlings, which showed uniformity in size and health condition were selected for this experiment. These plants were pruned to develop fresh foliage uniformly, and grouped into five groups representing (pH 5.0, 6.0, 7.0, 8.0, and 9.0). Each group was irrigated every other day with 1/18 strength of Hoagland solution adjusted to the pH level which the group is representing for 3 months. Then, 300 mg of young leaves were harvested and placed in liquid nitrogen immediately, and kept in −80°C for RNA extraction. The rest of the green foliage was harvested for mimosine and tannin quantifications.
(iii) Ten seedlings that showed uniformity in size and health condition were selected for this experiment. These plants were pruned to develop fresh foliage uniformly, and grouped into two groups representing nitrogen abundance and nitrogen deficiency conditions. The first group was irrigated every other day with a regular 1/18 strength of Hoagland solution adjusted to pH 7.0; whereas, the second group was irrigated every other day with nitrogen-free 1/18 strength of Hoagland solution adjusted to pH 7.0 for 3 months. Then, 300 mg of young leaves were harvested and placed in liquid nitrogen immediately, and kept in −80°C for RNA extraction. The rest of the green foliage was harvested for mimosine and tannin quantifications.

#### Mimosine Quantification

Mimosine was isolated as described earlier (Bageel and Borthakur, 2022). Mimosine concentration was determined by using high performance liquid chromatography (Waters 2650) with a C18 column and UV detection at 280 nm, using an isocratic carrier solvent of 0.02 M O-phosphoric acid and a linear flow rate of 1 mL per min for 6 min (Negi et al., 2014).

#### Tannin Quantification

Total tannin was isolated using the Folin-Ciocalteu’s method and quantified as described by Makkar (2003). Briefly, 1.0 g of leaves was placed in the oven for 16 h at 50–52°C to dry; then the sample was grinded to a fine powder. 200 mg of the grinded sample was added to 10 mL of aqueous acetone (70%) in a 50-mL falcon tube. The mixture was subjected to ultrasonic treatment for 5 min at 100 Watt, and then centrifuged for 10 min at 3,000 g at 4°C to remove plant debris.


(Total⁢Tannins=Total⁢Phenolics-Non⁢-⁢tannin⁢Phenolics)

#### Statistical Analyses

Plants used in this study were grown in pots in a greenhouse and organized in a completely randomized block design with five replications. For gene expression studies, qRT-PCR experiments were conducted with five biological and three technical replicates. For analyses of mimosine and tannin contents in giant leucaena grown with different treatment conditions, Student’s *t*-test was used with *p*-value of 0.05. Similarly, statistical significance for differences in gene expression levels between two sets of plants receiving separate treatments was determined using Student’s *t*-test with a cutoff *p*-value of 0.05.

## Results

### Bioinformatic Analyses

The results of quality analyses of the raw data from Illumina sequencing were checked by FASTQC tool, and e-value distribution of the unigenes (Table 1a and Supplementary Figure 2). The quality of the assembly was also judged by the length of unigenes, GC content, total assembled bases and N50 (Table 1b). The number of unigenes identified for giant and common leucaena were 130,825 and 116,332, respectively. On an average, there were around two transcripts for each unigene in the sequences for both giant and common leucaena. The N50 values, indicating the median contig lengths for the unigenes were 895 and 929 for giant and common leucaena, respectively, suggesting that the sequence assemblies were of reliable quality (Supplementary Figure 3).

**TABLE 1:** Representing (a) bioinformatics raw data quality, (b) gene assembly, and (c) functional annotation.

a								
**Subspecies**	**Raw reads**	**Raw bases**	**Valid reads**	**Valid bases**	**Valid%**	**Q20%**	**Q30%**	**GC%**

Giant	48,680,970	6.86G	47,927,522	6.70G	98.45	98.30	94.38	45.70
Common	43,049,726	6.07G	42,196,800	5.89G	98.02	98.27	94.32	46.95

b								

**Subspecies**	**Type**	**All**	**GC%**	**Minimum length**	**Median length**	**Maximum length**	**Total assembled bases**	**N50**

Giant	Transcript	268,663	43.29	186	565	13,399	232,314,065	1325
	Unigene	130,825	42.81	201	360	13,399	81,197,946	895
Common	Transcript	235,658	42.40	184	568	10,258	201,362,860	1,300
	Unigene	116,332	41.62	201	355	10,258	72,153,253	929

c								

					**Annotated unigenes**			
		
**Subspecies**		**NR**		**Swissprot**		**KEGG**		**Total**

Giant		56,623		48,450		17,944		65,321*
Common		48,983		43,895		14,883		58,848*

*Some of the genes were common to all three databases.

These unigenes were annotated using group blast against sequence databases, including nr, Swissport, GO, and KEGG. For giant leucaena, the annotation with the nr database had the highest aligned unigenes (56,623 unigenes), followed by the annotation with the Swissport database (48,450 unigenes), followed by the annotation with the KEGG database (17,944 unigenes). With the three databases combined, a total of 65,321 unigenes were annotated. For common leucaena, the annotation with the nr database had the highest aligned unigenes (48,983 unigenes), followed by the annotation with the Swissport database (43,895 unigenes), followed by the annotation with the KEGG database (14,883 unigenes). With the three databases combined, a total of 58,848 unigenes were annotated (Table 1c).

**FIGURE 2:**
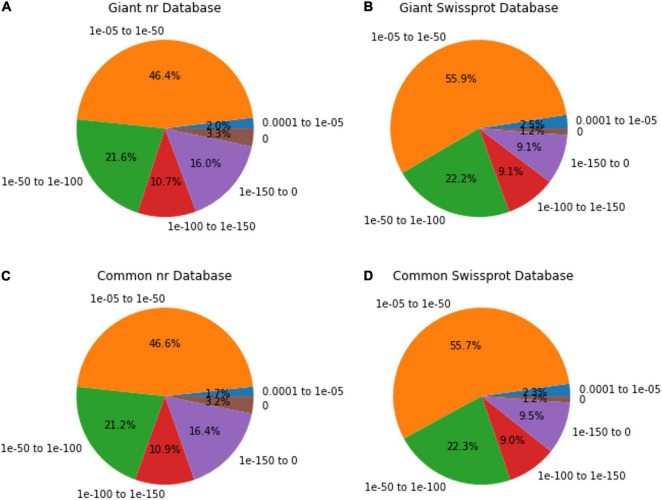
E-value distributions of annotated giant and common leucaena unigenes. The E-values of the highest-scored BLAST hit were identified for each unigene. **(A)** Aligning giant leucaena against the nr protein database, **(B)** aligning giant leucaena against the Swissport protein database **(C)** aligning common leucaena against the nr protein database, and **(D)** aligning common leucaena against the Swissport protein database.

Among the unigenes annotated in the nr and Swissport databases for giant leucaena, 51.6 and 41.6%, respectively, had an E-value < 1.0E-50. For common leucaena, the unigenes annotated in the nr and Swissport databases, 51.7 and 42%, respectively, had an E-value < 1.0E-50. Transcriptome sequences with very small values are desirable because small E-values indicate high accuracy. On the other hand, high E-values reduce accuracy and increase probability for error. In this study, 3.3% of the transcriptome sequence in the nr database comparison showed an E-value of zero. The highest E-value of 1e-04 to 1e-05 was observed for only 2% of the transcripts in the nr database comparison. The rest of the giant leucaena transcripts had intermediate E-values between these lowest and highest values. The common leucaena transcripts also showed similar E-values (Figure 2).

The number of transcripts found for different genes in giant and common leucaena (Supplementary Table 1) were different; some of the genes had high numbers of transcripts in giant leucaena and low in common leucaena and vice versa. For example, mannose/glucose-specific lectin-like protein isoform X2 had 11,843 transcripts in giant leucaena compared to only 105 transcripts in common leucaena. On the other hand, abscisic acid receptor PYL6 had only 24 transcripts in giant leucaena compared to 723 transcripts in common leucaena. This suggests differential expression of the genes in the two subspecies. This is further supported by the observation that the transcript per million (TPM) for the same genes also vary greatly between giant and common leucaena (Supplementary Table 1). Giant leucaena genes which are up-regulated or down-regulated relative to common leucaena are shown in Supplementary Table 1.

### Expression of Genes Related to Fodder Quality

From the transcription analysis the nucleotide sequences for two genes for mimosine metabolism, five genes for tannin biosynthesis in giant leucaena were obtained (Table 2 and Supplementary Table 2). It is known that mimosine in the leucaena foliage is degraded under certain environmental conditions. Therefore, the gene sequence for mimosinase was also selected for gene expression studies. Previous studies show that the mimosine contents in the leucaena foliage change under different environmental conditions such as soil salinity, pH and nitrogen content (Honda and Borthakur, 2021; Bageel and Borthakur, 2022). Similarly, the tannin contents of the leucaena foliage are influenced by a number of environmental factors. The effects of some of these environmental factors on expression of genes for mimosine biosynthesis and degradation, and tannin biosynthesis were determined by qRT-PCR analyses.

**TABLE 2:** Sequences and primers of the genes of interest.

Gene	Sequence	Length	Forward primer	Reverse primer
Mimosine synthase	GTTTTGAGCTGGACTTCACGGAGCTGCCCTAGATCGCTGTCCATTGT TATCGGCTGCTTCGTGGAGGCAGGGTTAGAGAGTAAGCTATCACCAT GGCAGTGGAACGGAACGGAATTGCCAATGATATTACAGAATTGGTTG GCAAAACCCCATTAGTATATCTTAATAAAGTTGTGGATGGCTGTGTGG GCCGGATTGTTGCCAAACTAGAGTTTATGGAACCCCTCTCTAGTGTC AAAGACAGGATCGGCTATAGTATGATTGCTGATGCAGAGGAAAAAGGT CTTATCACGCCTGGAAAGAGTGTCCTTATTGAACCAACAAGTGGTAATA CTGGCATTGGATTAGCCTTCACGGCAGCAGCAAAAGGTTACAGGCTT ATTATTGTAATGCCTGCTTCAATGACTCTTGAAAGAAGAATGGTTCTGA AAGCTTTAGGGGCTGAGTTGGTGCTGACGGATCCTGAAAAGGGAATA AGAGGTGCATTTCAGAAAGTTGAAGAAATATATGCTAAGACACCTAATT CCTACGTACTTCAACAGTTTGAAAATCCTTCCAATCCCAAGATGCATT ATGACACTACTGGCCCAGAGATTTGGAAAGCCACTGAAGGGAAAATTG ATGTACTTGTAGTAGGGTTAGGTACTGGTGGCACAATAACAGGTTCTGG CAAATTTCTTAAAGAACAAAATCCCAATATTAAGGTTTGTGCTGTTGAAC CTGCTGAAAGTCCCGTGCTCGTTGGTGGACAGCCTGGTTCTCATGG GATTTATGGGATTGGTGCTGGTTTTATACCTAAGGTGGTGGATGTCAGT CTTCTTGACGAAGTTGTTCATGTAACAACCTCTGAAGCCATTGAAACTG CAAAGCTTCTTGCAGTGAAAGAAGGCCTATTTGTGGGAGTTTCATCTGG AGCTGCAGCAGCCGCTGCAATTAAGGTTGCAAAACGACCAGAAATGG CTGGAAAGCTTATTGTAACGATTTTTCCAAGCTGCGGTGAGAGATATCTG TCGTCTGCGTTGTTTGAGTCGATCAGAGAAGAATCTGAGAACATGACTT TTGAGCCATAAAATTTGGATTTCAAGGCTTTCAAGCTACTTTAGTTCTGAT ACTGTTGCAATGCCGGTGGCTTTCGTGGTTGTGATTCTGAAATAAAAATT CGTAGAAAAGTTGTAGCAAAAAACCCCCAAACTTTTAGTGTCATATTTGG TTGTCTTTGACAGTTTGATTCTGTTTGAATAATGTTGGTTATAATCGGCAAA AAAAAAATGTAACAAAAGAAAATAGTGTGGTTCTTGAACATTATTTAATCAA GATGGAACGTTTTTCTGTTTTACTATAATCGGCAGATCGGAAGAGCACAC	1,364 (102)	5′-TCCTTCCAATC CCAAGATGC-3′	5′-CCACCAGTACC TAACCCTACTA-3′
Mimosinase	CTGGCTTTTAATTGTCCAGGTGAAGTTGTGGTTTGCTACACGGCTAATCG AAGAGCATAAGCAAAACCTCTATAGCTCTTTCTTGATCTTCGGAGATGGC TTTGTCATCAACTTTTCTCAACCCTCTCGTTTCTTCGGTCACTGTTAACCC TCACCCTAAGATCACAAATGGAAAGGGGTTCAGAGTAAATTGCTTAATCA GAACACAGCAGACTGTTATCAAAACGGATGCGAAGGAGAATGCTGCGG TGCTTACACCAGGAAAAAAAGTGGAAAAAGAACCAAGTGTGTCTACAGT GTTAGCGAATTACCATGCTGACTGGGATCCTTTCGAGGCAACTTCTACA CCAATTTACCAAAGTGCTACTTTCAGAATGAAAAATGCAACCGAATATAAT GAGTATTACTATTGTAGAGTTGGAAATCCTACCACGAGTACTTTGGAAAA GATATTAGCGGAGATTGAACACGCAGAATATGTCACCTGCTTCACTAGT GGAATGTCTGCTTTGACTGCTGTTTGCGAACTTGTTTCTCCTGGGGATG AAATCCTTACTGTAGAGGATATCTATGGGGGTTCATATGGCTTCATAGAA AATCTAATGGTCAGAAAGTCAGGAGTCACAGTGAAGAGAGTAGATACA AGCAAACTAGAGAACGTGAAAGCTGCTATTACTGATAAGACTAAGTTG GTGTGGCTAGAGTCTCCCAGTAATCCTCAGCTAAAAATCTCTGATATCC GAGAAATAGCCAAGATAGCCCATGCACATGGTGCTATTCTATTTATTGA CAATTGTATAATGTCTCCTCTGTTAAGTCATCCATTGGAATTGGGAGCA GACATCGTTATGCATTCAGCTACAAAATTTATTGCTGGAAACAGCAGT TGCATGGCTGGCAGTCTTGCCACGAACAACAAAGAGTTGGCCGATCT ACTACTCTCTTACCGAAGTGCGACGGGCTGTGGGTTGTCTCCACAGG ACGCCTGGATTTGTTTGGAAGGAATCAAAACATTGCCCCTGCGAGTTG AGGAGAAACAGAAAAATGCACTAACTGTTGCTAATTATCTTCACAACAAT CCTAAAATAACAAAAATAAACTATCCTGGTCTTCCCGATAATCCTGGATA TGAGTTGCATAAATCTCAGTCAAAAGGGCCAGGATCTGTCATGAGCTTT GAAACAGGCTCACTGCCACTCTCAAAACAAATCGTTGAAGATACTAAA TTCTTCAGCAAGATTGTTGGTTTTGGTGGTGTTGGTTCCGCCATATGCT TGCCTTGGTATACATCCCATAAGGCCATTCCGGAGCCAGAGAAAATTAG AATGGGTATAACTAAAGATCTTGTACGTGTCTCTGTGGGAATTGAGGAT GTTCAAGATCTCATTCAAGATCTGGATAATGCAATGTCAACTCCTACGTT CTGAAGTTCGTATGTCAAACGTACTGCTTATTTGCTTCAAATAATGAGATC GATCGAGGTTGCTGATGTTTTGTTGACTCTATTGCCTCTGTTATCTCTTTG CATTGTATTTGGCTGTAACAGTTTGGAACAGTACTAAGCTTATTATTGAAG ATAATCCTATTTTTCATTTGGCACTGGTGTCTTTAAATAGTTTACCTTCCCC TCTCTCTCTCTACCTTCTTGCTTAATTTATTGCATTTTACGTTTCGCGAC GATAATTGTATATAATGATGATG	1,701 (98)	5′-CTGCGAGTTG AGGAGAAACA-3′	5′-ATCGGGAAGA CCAGGATAGT-3′
Chalcone synthase (CHS)	CTCGCAATTTTCTGCAACTTCACATCAAACAAATCCCAATATCTG TAATTAATCTTGTTCATCAAAATGGTGAGCGTTGATGAGATCCGC AATGCCCAGAGAGCAGAAGGGCCGGCCACCGTGATGGCCAT CGGAACGGCCACTCCACCGAACTGTGTTGATCAGAGTACATAC CCTGATTACTACTTCAGAATCACTAAAAGCGAGCACAAGACCG AGCTCAAGGAGAAATTCAAGCGCATGTGCGAAAAGTCAATGA TCAAGAAGAGATACATGCACTTGACGGAGGAGATCTTGAAGG AGAACCCAAACGTGTGTGAGTATATGGCTCCTTCTCTGGATGCA AGGCAAGACATGGTGGTCGTGGAAGTCCCCAAGCTCGGCAAA GAGGCCGCCACCAAAGCCATCAAGGAGTGGGGCCAGCCCAA GTCTAAGATCACTCACCTCATCTTCTGCACCACCAGCGGCGTCG ACATGCCCGGCGCCGACTACCAGCTCACCAAGCTCCTCGGCC TCCGCCCCTCCGTCAAGCGTTACATGATGTACCAGCAGGGCTG CTTCGCCGGCGGCACGGTCCTCCGCCTGGCCAAGGACCTGGC CGAAAACAACAAGGGAGCTCGCGTCCTCGTCGTCTGCTCCGA GATCACCGCCGTCACTTTCCGTGGACCCAGCGACACCCACCTG GACAGCCTCGTGGGCCAAGCCCTCTTCGGAGACGGTGCGGCCG CCGTCATTGTCGGCTCTGACCCCCTCCCTGTGGAGAAGCCTCTG TTTGAGCTTGTGTGGACGGCTCAGACCATTTTACCGGACAGTGAA GGAGCCATTGACGGACACCTCCGCGAGGTTGGCCTCACCTTCC ATCTACTCAAGGACGTTCCCGGGCTGATCTCGAAGAACATAGAG AAGGCATTGGTGGAAGCGTTCCAGCCACTGGGGATATCGGATT ACAACTCCATCTTCTGGATTGCTCACCCGGGAGGGCCTGCCAT TCTTGACCAGGTAGAGGCGAAGCTGAGCTTGAAGCCAGAGAAG ATGAGGGCCACAAGGCACGTGCTGAGTGAGTATGGGAACATGT CGAGTGCTTGCGTGTTGTTCATTTTGGACGAAATGAGGAGGAA GTCTGCAGAAGATGGGCTCAAGACCACTGGTGAAGGACTTG AATGGGGTGTTCTGTTTGGATTCGGGCCTGGGCTCACCGTTGA AACTGTTGTTCTTCACAGTGTGGGTACTAACTAATTGGTCCAA TAGAACGCTGAAAATGAACGGGGAGGCCCAATCGTGAATAAC TTATACTTCTTCTTCCACTTTTTGGGAGACTCTTGGATGTTTTTAT TATTTTTTTAGCATGTACTGTTTATTAAAAGTTAGCCTGTCTTTAG TGTGGTCTGTGTAGAGTGCAAAAGAGTTTGCAATATAAAAATAC GATATTGATATCCAAGCAAGATC	1,446 (95)	5′-GGGCTGATCT CGAAGAACATAG-3′	5′-GGGTGAGCA ATCCAGAAGAT-3′
Flavanone 3ß-hydroxylase (F3H)	CCTCCCCTTCCTTTTCTCCATCTACTCTCACTCCGTCTTCTTCA ATCTTCACTCGTCACCGCACCACTCAGCCCCATGGCGCCCG CCAAGACTCTCACTTCCCTTTCCGACGAGAACACCTTCGACT CTCGCTTCCTCCGCGACGAAGACGAGCGCCCCAAGGTCGCTT ACAACCAATTCAGCAATGATATCCCCATCATCTCTCTCGCCG GCATTGATGACGTCGACGGTCGCCGCGCCCAGATCTGCCA GCAGATTGTTCAGGCTTGCGAGGATTGGGGCATCTTCCAG GTCGTCGATCACGGCGTCGACACTAAACTCGTCTCCAGCAT GACCAGTCTCGCCAAGGAGTTCTTCGCCATGCCCCCCGAA GAGAAGCTCCGCTTCGATATGTCCGGCGGCAAGAAGGGCGGT TTCATCGTCTCCAGCCATCTCCAGGGGGAGATGGTGCAGGATT GGAGAGAGATCGTGACTTACTTCTCGTACCCAATTAGGAACAG GGACTACTCCAGGTGGCCGGACAAGCCGGAATCCTGGAGGA AGGTGACGGAGCGATACAGCGAGGACCTGATGGGGTTGGCAT GTAAGCTGCTGGAGGTTCTGTCGGAGGCGATGGGTCTGGAGA AGGAAGCCCTGACGAAGGCGTGCGTGGACATGGACCAGAAGG TGGTGGTGAATTACTACCCGAAATGCCCGCAACCGGACCTCACC CTCGGACTCAAGCGTCACACTGACCCGGGAACCATCACCCTCC TCCTTCAGGACCAGGTCGGGGGACTCCAGGCCACCAGGGATAA CGGCAAGACGTGGATCACGGTCCAGCCTGTGGAAGGCGCTTTC GTTGTCAATCTCGGGGATCACGGTCATTTCCTGAGCAACGGGAGG TTCAAGAATGCGGATCACCAGGCGGTAGTGAACTCGGAGCACAGC CGTCTGTCGATAGCGACTTTCCAGAACCCGGCGCCAGATGCGATAG TGTACCCGCTGAAGATTAGGGAGGGAGAGAAGTCGGTGATGGAG GAGCCGATAACGTTCGCGGAGATGTACAGGAGGAAGATGAGCAAG GACCTGGAGCTGGCGAGGTTGAAGAAGATGGCTAAGGAGGAGAAG CAATTGCAGGAGATGGAGAAGGCCAAATTGGAAGGGAAGCCCATCGA GCAGATACTTGCCTGATCGACCGAATTTCGATATGTTTCATTTAGTACG TATTAGTTGGGTGGTTATGCTATAGGTGACGTAAACAACAATATGCAGTT			
	GGCGTTGGCAGTGTTTTGCTGGATTTATTTATGGGTCGTGTGTTTTTCC CTGTAAACCACGTTACTTAGTCTTAAACATGAACAAATAAGTTGTGCCC CCCTCATCGTTTTCTTTCCGGTTATCCTTTTGTAACGTATTATATTCCAAA TAGAATTAAGAGAC	1,419 (116)	5′-CCCAATTAGGA ACAGGGACTA-3′	5′-GAACCTCCAGC AGCTTACAT-3′
Leucoantho-cyanidin reductase (LAR)	GAGAGAGAGAGAGAGAGAGAGAGAGAGAGAGCATACTGAGACCTTTAT CATTTGACTTTTTCGTCTTTTCATCTTCGTCGTCGTTGTCGTTGTCAACAT GACTGTGTTGCCTGCTGTTGTTGGGAAGAAGGACCGGGTGCTCATTGC TGGAGCCACAGGTTTCATCGGTCAGTTCGTGGCGGAGGCGAGCCTGG CAGCGGGTTACGCCACCTATTTGCTCGTACGGCCGGGACCCTTCAGCC AATCCAAGTCTTCCCTTATGAAAGGGCTCCAAGAGAAAGGTGCAGTTGT CATTAATGGGGCGATAAATGAGCAGGAATTAATAGAGAAGATTCTAAGAG ATTATGAGATAGAGATTGTGGTATCAGCTGTGGGAGGCAGAAACTTGCTT GACCAGCTCGTCCTGGTGGAAGCCATGAAATCTGTTAAAACTATCAAGA GGTTCATGCCATCGGAATTCGGGCATGACGTGGATCGGGCTGACCCCG TGGAGCCGGGTTTGGGGATGTACAAACAGAAGCGTCTGGTGAGGCGC GTGGTGGAGAAGTCTGGGATCCCCTACACTTACATCTGCTGCAACTCCA TCGCTTCTTGGCCTTACTACGATAACTGCCACCCTTCCCAGAGTCCTCCG CCCTTGGATCAATTGCACATCTACGGTGATGGCACTGTCAAAGCGTAC TTCGTTGGTGGACCTGACATTGGGAAATTCACAATGAAAGCTGCAAGTGA TATCAGAGCCCTGAACAAGAGCCTCCATTTTCAACCAGCGGCCAACTA CTATAACATGAGCGAGCTCGCTTCCATGTGGGAAACCAAAGTTGGTCG CACCATTCCCAGAGTCATCATCACTGAAGATCACCTCCTTGCTGTTGC GGCTGAGAAAGTTGAGCCGAGGAGCTTTGTGGCATCGTTTACACATG ACATCTTCATCAAAGGGTGCCAGATCTTCAAGCCGGACGGGCCAGG TGATGTGGAAATCGGTTCACTGTATCCTGAAGAACAAATAAGGAGGTT GGAGGATTGCTTTCAGGATTTTGTTCTGATGATACCGCCGGCGGAAAC CAAGAAGACGACAACAACGGAGCTTAACTGTACTGATAACGGCGTTG CGACCAACAACACCGGTGACAGCAACGACACTACTGCCACCGCCAC CGCCCCCACAAACTCATCGGCTGTGGTAAAGCCGGTGCCAATCACGG CTATGTGTTGATGATCGATCAATCATGATGATGATGATGAAATGAGTTCA TATGATATTTAGTAGTATTTGATCTCTCTCTCTCTCTGTTTTACCATTATTA TTGGGAATTTAATTTGTGAGACGGGAATCTTGTTTATTCATATGGTGGTT GGATGTTGCTGGCAGTGTTTGAACAAGAATAAACCCATATGAGTAATAT AATATTTTAAGGCTATTTTGTTCTTGTAGTATCCCACAATTGATATCTCTT CACATCAATTGATGCTGTAATAAATTTTTATTAAAATTCTTATATGACT GTTCCCAAATACGC	1,520 (112)	5′-AAAGGGCTCCA AGAGAAAGG-3′	5′-CCACAGCTGA TACCACAATCT-3′
Dihydroflavonol reductase (DFR)	ATTCAATTGACTTTTCGTCCCCAATAACACCCTCCTCCTCTTTACCCTT TATATTATATTGCTCATTCCGGCCAGTTCCGAGAAAGAGACTGCAGAGT GAGAGATAACCATGGGTTCAGTGTCTGAGACTGTTTGTGTCACTGGC GCTGCCGGCTTTATCGGTTCATGGCTCATCATGAGACTCTTGGAGCG CGGCTACACCGTTCGAGCCACCGTGCGCGACCCTGCTAACATGAAG AAGGTGAAGCATTTGCTACAGCTGCCAAAAGCCGACACACACCTCAC TCTGTGGAAGGCCGACCTTGCCCACGGGGGAAGTTTCGACGACGCC ATCAATGGCTGCACTGCAGTTTTCCATGTCGCCACCCCCATGGATTTC GAATCCAAGGATCCTGAGAATGAAGTGATACAACCAACGATCAATGGT TTATTGAGCATCATGAAAGCATGCCTCAAGGCCAAAACTAGAAGATTG ATATTCACGTCCTCCGCCGGAACTGTGGATGTCACAGAGCACCAAAA ACCTGTGTTTGACGAGACTTGTTGGAGTGATGTCGAGTTCTGTCGCAG AGTTAAGATGACTGGTTGGATGTATTTCGTTTCAAAAACACTGGCGGAG CAAGAGGCATGGAAATTTGCCAAAGAGAATAACATGGATTTCGTCTCTAT AATTCCTCCTCTCGTTGTTGGCCCCTTTCTCATGGATTCAATGCCTCCC AGTCTCATTACCGGCCTTTCTCTTATCATCGGGAACGAAGCACATTATCC AATCATAAAGCAAGGTCAATTTGTTCACTTGGATGACCTCTGCATGGGT CACATATTCTTGTTAGAGCATCCTGAAGCAAAAGGAAGATACATGTGCT CTTCCCATGATGCCACCATTCATGACGTTGCAAGAATGCTTAGTAAAAA TTACCCTGAATACAATATCCCCACCAAGTTTAAAAACATCCCAGATGAG TTGGAGATCATCCACTTCTCTTCCAAGAAGATTAAAGATTTGGGATTCC AGTTTAAATACAGCTTAGAGGACATGTTCACAGCAGCTGTTGACACCT GCAGGGAAAAAGGGCTTCTTCCAAAGCCTGTTGCAGAAATCCCTGTC AATGGCACCGCAGCTGATCAGAAGTGATTTGGGAAATGTATCCTCGTG TTCGCCATAACCTTTGGCTCTTCTATATATTTAGTTTTTGGTGTTCTAAA TTACGGCTTGGGATATTTATGGGGTCTGGAAATAAGAATGGCTTAGGAC CCGATGGCGGCGGAG	1,270 (204)	5′-CGAGTTCTGTC GCAGAGTTAAG-3′	5′-GCTTCGTTCCC GATGATAAGAG-3′
Anthocyanidin reductase (ANR)	GGCAGTCAGTGGCTACAGAAGAGAGCGCCGGTAGATAGGAGGTGTG ACTGTTTGTGGGATTTGGAATCGTAGATTTGGATTCCCAAGTCTGGGTA GCTGAATCCTAACGAAAAGGGAAAAAATCAAGCCTTCTTCTTGGTGGC GTCGTTGGTCATCACGTGCTCACCTAAGTCCCCTTCCACCTCTGTTG TTCTCACTCAACTCAGCACCCACAAAGCCACCTTATATTAGTTCTGCC GTCTCCCTGCATCAACCAATCGGGAAGAAGAAGAAGAAGATGATGAAG GCGTGCGTAGTGGGAGGAACCGGGTTCGTCGCTTCTGAGCTCGTTA AGCAGTTGCTTCTCAAGGGATATGCTGTTAATACAACTGTTCGAAATCC AGATGATCGCAAAAAAATTTCCCATCTCATAGAACTGCAAAAATTGGG AAAATTGAACATATTTGGAGCAGATCTGACTGAAGAAGACAAATTTGA TGCCCCTATTCTTGACTGTGAACTTGTCTTCCATGTTGCTACTCCTG TGAACTTTGCTTCTGAAGATCCTGAGAATGATATGATCAAACCAGCA ATCCAAGGCGTATTGAATGTCTTGAGAGCATGTGCGCGAGCAAAA GCAGTTAAACGAGTGATCTTGACATCTTCAGCAGCTGCTGTCACA ATAAATGCATTAGAAGGGACTGGTCTGGTTATGGATGAAAACAACT GGACTGACGTTGATTTCTTGAGCACTGCAAAACCACCTACTTGG GGATATCCTGCCTCCAAAGCACTAGCAGAGAAGGCTGCATGGAA ATTTGCCGAAGAAAATCATATAGATCTCATCACTGTGGTACCTACTA TGATGGGTGGTGCTTCTCTCACTCCAGAAATCCCAGGCAGTCT TGGTCTCGCCATGGCCCTAATTACAGGCAATGATTTCCTCATAAA TGCTTTGAAAGGCATGGAGATGTTGTCAGGTTCAATATCCCTTGC TCATGTGAAGGATGTTTGCCGAGCACACATATTTTTGGCTGAG AAAGAATCAGCTTCTGGTCGATATATTTGTTGTGCTCACAACAC AAGTGTTCCTGAACTTGCAAAGTTCCTTAGCAAACGATACCCT CAATACAAAATCCCAACCGAATTTGATGGCTTTCCCTCTAAGGC CAAGTTGGTAATCTCTTCAGAGAAGCTTATCAAAGATGGGTTCAAT TACCAGTTTGGGGCAGAACAGATCTACGACGAGACTGTGGAGTAC TTCAAGTCCAAGGGAGCCCTTAAAAATTGAATTAGTAGAGGTTCAA AGCTCTAGCAGAAGCTCCACTAGTTATAATTATGTAATGATGATAAAA ATGTGGAGCGGCAGTTCAGTGTGATATATACTGAACTAGTATTTTTAT ATGATGGTATTCTACCCTTCCACCTTGTCCCCTGTAATGCACTTTCT GATATGTTAGTGCACTTTCTGATATGTTAGTGAAAAGCCGAAGAAGA AAAGAAAGAGAAGATTATTGGAAAATAAAAAAATCCAACG	1,516 (481)	5′-CGTCGCTTCT GAGCTCGTTA-3′	5′-CCATGCAGCC TTCTCTGCTA-3′
				

### Expression of Mimosine Synthase and Mimosinase Under Different Salinity Conditions

**FIGURE 3:**
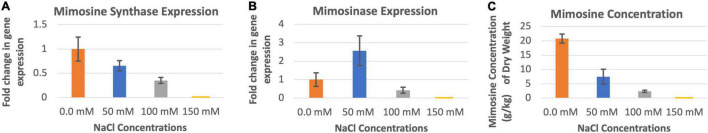
Changes in the gene expression for mimosine synthase **(A)** and mimosinase **(B)** under four different salinity levels. The changes in mimosine production under different salinity conditions **(C)** are also shown. Mimosine production was highest at 0.0 mM salt condition. Therefore, 0.0 mM NaCl was considered as the reference for comparisons; and thus, the expression in panels **(A,B)** values at this salt concentration is one. The error bars in panels **(A–C)** indicate ± SE (*n* = 15, 5 biological each having three technical replicates).

The highest level of mimosine synthase activity was observed in the absence of salt in the soils (Figure 3A). At 50 and 100 mM salt concentrations, the expression of mimosine synthase decreased progressively. Leucaena could not tolerate and grow in 150 mM salt concentration and therefore data could not be recorded. Thus, the presence of salt appears to inhibit mimosine synthase activity. On the other hand, mimosinase expression did not appear to have a direct relationship with salt concentrations; its expression was highest at 50 mM salt concentration and lowest at 100 mM salt concentration (Figure 3B). Mimosinase expression was intermediate in the absence of salt in the soil. Thus, it appears that some amount of salt is inducive for mimosinase activity while too much salt is inhibitory for mimosinase activity. Mimosine concentration in the leucaena foliage was negatively correlated (R^2^ = 0.88) with salt concentrations in the soil (Figure 3C). However, mimosine synthase activities had strong positive correlation with mimosine concentrations in the foliage (R^2^ = 0.78). On the other hand, mimosine concentration had no correlation with mimosinase activity in the foliage (R^2^ = 0.11).

### Expression of Mimosine Synthase and Mimosinase Under Different Soil pH Conditions

**FIGURE 4:**
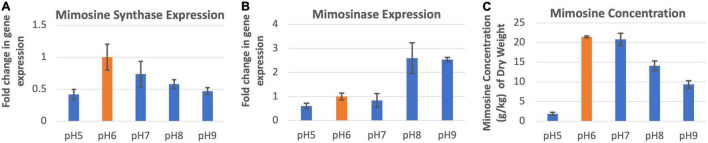
Changes in the gene expression for mimosine synthase **(A)** and mimosinase **(B)** under five different pH levels. The changes in mimosine production under different pH conditions **(C)** are also shown. Mimosine production was highest at pH 6.0. Therefore, pH 6.0 was considered as the reference for comparisons; and thus, the expression in panels **(A,B)** values at this pH is one. The error bars in panels **(A–C)** indicate ± SE (*n* = 15, five biological each having three technical replicates).

The expression of mimosine synthase in the leucaena foliage was found to be highest at pH 6.0 and lowest at pH 5.0 (Figure 4A). The foliage of plants grown at pHs 6.0–9.0, showed progressively lower levels of mimosine synthase expression activity. Mimosinase expression in the leucaena foliage was relatively low at pHs 5.0, 6.0 and 7.0. and was much higher at pHs 8.0 and 9.0 (Figure 4B). Thus, alkaline pH was favorable for mimosinase expression. Mimosine concentrations in the leucaena foliage were highest at pHs 6.0 and 7.0 (Figure 4C). Mimosine concentrations in the leucaena foliage decreased with the increase in the soil pH above 7.0. Mimosine concentration in the foliage appears to be directly correlated with mimosine synthase expression (R^2^ = 0.78).

### Expression of Mimosine Synthase and Mimosinase Under N-Abundance and N-Deficiency Conditions

**FIGURE 5:**
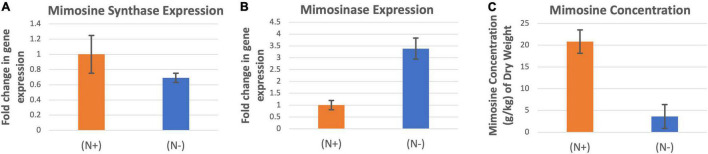
Changes in the gene expression for mimosine synthase **(A)** and mimosinase **(B)** under nitrogen abundant (N +) and nitrogen deficient (N-) conditions. The changes in mimosine production under these two conditions **(C)** are also shown. Mimosine production was higher at (N +) condition. Therefore (N +) was used as the reference for comparisons; and thus, the expression in panels **(A,B)** values under (N +) condition is one. The error bars in panels **(A–C)** indicate ± SE (*n* = 15, five biological each having three technical replicates).

The expression of mimosine synthase was significantly higher in the leucaena foliage under nitrogen abundant condition than in nitrogen deficiency condition (Figure 5A). On the other hand, mimosinase expression was significantly higher under nitrogen deficiency condition than in nitrogen abundance condition (Figure 5B). Mimosine concentration in the foliage was much higher when the plants were supplied with combined nitrogen compared to nitrogen deficiency condition (Figure 5C).

### Expression of Five Tannin Biosynthesis Genes Under Different Salinity Conditions

**FIGURE 6:**
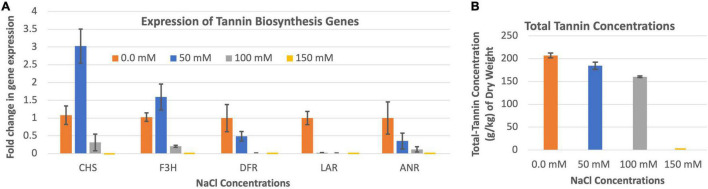
Changes in the gene expression for tannin biosynthesis including chalcone synthase (CHS), flavanone 3ß-hydroxylase (F3H), dihydroflavonol reductase (DFR), leucoanthocyanidin reductase (LAR), and anthocyanidin reductase (ANR) **(A)** under different salinity conditions. The changes in tannins production under different salinity conditions **(B)** are also shown. Total tannins production was highest at 0.0 mM salt condition. Therefore, 0.0 mM NaCl was considered as the reference for comparisons; and thus, the expression in panel **(A)** value at this salt concentration is one. The error bars in panels **(A,B)** indicate ± SE (*n* = 15, five biological each having three technical replicates).

The expression of CHS and F3H were highest when the leucaena plants were grown in the presence of 50 mM salt, while the expressions of DFR, LAR and ANR were highest when the plants were grown in the absence of salt (Figure 6A). Tannin concentration in the leucaena foliage was also highest when the plants were grown without adding any salt to the soils (Figure 6B). The tannin concentrations progressively decreased with addition of 50 and 100 mM salts to the soils. Thus, tannin concentrations in the leucaena foliage appear to be negatively correlated with the amount of salt present in the soils (R^2^ = 0.79). CHS, F3H, and DFR had weak correlations with tannin concentrations in the foliage (R^2^ = 0.34, 0.52, and 0.47), respectively. On the other hand, tannin concentration had strong correlation with LAR and ANR gene expressions in the foliage (R^2^ = 0.74 and 0.88), respectively.

### Effects of Soil pH on Tannin Biosynthesis

**FIGURE 7:**
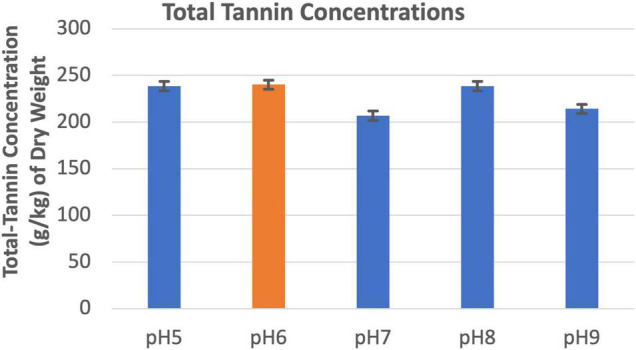
Changes in total tannins concentrations under different pH conditions. The error bars in indicates ± SE (*n* = 15, five biological each having three technical replicates).

Soil pHs between 5.0 and 9.0 did not appear to affect tannin concentration of the leucaena foliage (Figure 7).

### Expression of Five Tannin Biosynthesis Genes Under N-Abundance and N-Deficiency Conditions

**FIGURE 8:**
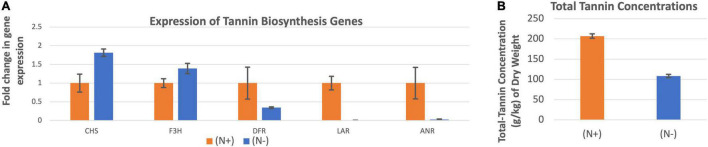
Changes in the gene expression for tannin biosynthesis including chalcone synthase (CHS), flavanone 3ß-hydroxylase (F3H), dihydroflavonol reductase (DFR), leucoanthocyanidin reductase (LAR), and anthocyanidin reductase (ANR) **(A)** under nitrogen abundant (N +) and nitrogen deficient (N-) conditions. The changes in tannins production under these two conditions **(B)** are also shown. Total tannins production was higher at (N +) condition. Therefore (N +) was used as the reference for comparisons; and thus, the expression in panel **(A)** value under (N +) condition is one. The error bars in panels **(A,B)** indicate ± SE (*n* = 15, five biological each having three technical replicates).

The expression of chalcone synthase and flavanone 3β-hydroxylase were higher when the leucaena plants were grown without providing combined nitrogen, whereas the expressions of dihydroflavonol reductase, leucoanthocyanidin reductase and anthocyanidin reductase were higher when the plants were grown with added combined nitrogen (Figure 8A). Overall, the combined expression of all tannin biosynthesis genes resulted in higher tannin concentration under N + condition compared to the N- condition (Figure 8B).

## Discussion

The concentrations of secondary metabolites such as mimosine and tannin are important aspects of nutritional quality in the leucaena foliage. In the present study, mimosine and tannin concentrations of leucaena foliage were studied under different salinity, nitrogen availability, and pH conditions with a goal to identify optimum conditions to obtain more nutritious fodder. Other major considerations for forage nutrition are protein content, palatability, digestibility, and total biomass productivity of the foliage. The present study is limited to only mimosine and tannin concentrations in the leucaena foliage because their presence reduces the quality of the fodder.

**FIGURE 9:**
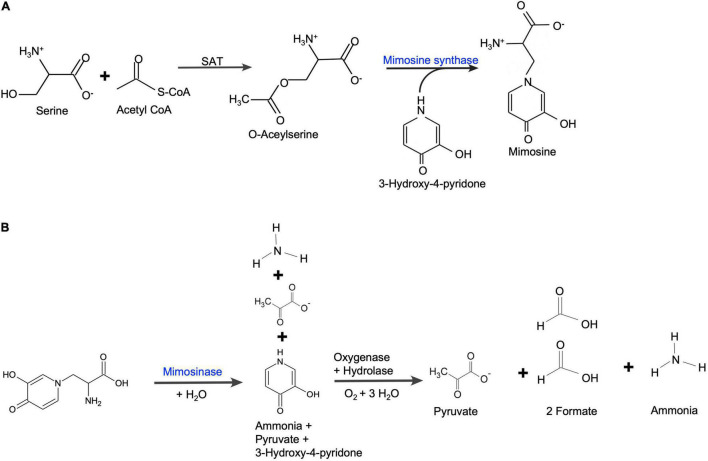
**(A)** Mimosine biosynthetic pathway in leucaena. Serine acetyltransferase (SAT) catalyzes the formation of *O*-acetylserine from acetyl-CoA and serine. *O*-acetylserine (thiol) lyase (mimosine synthase) catalyzes mimosine biosynthesis from *O*-acetylserine and 3-hydroxy-4-pyridone [based on Yafuso et al. (2014)]. **(B)** Mimosine degradation pathway in leucaena. Mimosine is degraded into pyruvate, formate and ammonia in two steps. First, mimosine is degraded into one molecule each of ammonia, pyruvate and 3-hydroxy-pyridone by mimosinase (Negi et al., 2014). Then, 3-hydroxy-4-pyridone is further degraded by a dioxygenase and a hydrolase into one molecule of ammonia, one molecule of pyruvate and two molecules of formate (Awaya et al., 2005).

The expression of two genes for mimosine metabolism, mimosine synthase and mimosinase, were studied. The positions of mimosine synthase and mimosinase in the mimosine metabolism pathways are shown in Figure 9. There was a direct correlation between mimosine content and the expression of mimosine synthase in the foliage, whereas there was a negative correlation between mimosine content and mimosinase expression. Thus, mimosine concentration in the foliage at any stage is the result of both synthesis and degradation. The mimosine synthase expression decreases with increase in salt concentration. However, neither low nor very high concentration of salt is favorable for mimosinase expression. Therefore, mimosine concentrations in the foliage is mostly a function of mimosine synthase activity. Recently, mimosine has been described as a “stress response molecule” that is produced in high quantities during favorable environmental conditions and degraded under stress environments such as drought and other nutrient-limited growth conditions (Honda and Borthakur, 2021). Under unfavorable growth conditions, mimosine in the leucaena tissues are converted to produce ammonia and pyruvate that are used in synthesis of other compounds essential for survival.

Among the various pHs tested in this study, the most favorable pH for mimosine biosynthesis was found to be 6.0. Around this favorable soil pH, mimosine is accumulated in the leucaena foliage. The mimosine concentration in the leucaena foliage decreases with increases in pH from 7.0 to 9.0. Under such unfavorable soil pH conditions, mimosine is degraded, and the byproducts are used as sources of nitrogen and carbon for synthesis of other more essential compounds. Similarly, mimosine synthase expression also decreases with increases in pH. On the other hand, mimosinase expression was low under acidic or neutral pH but high under alkaline pH conditions.

Mimosine synthase expression is also positively influenced by high nitrogen availability conditions. On the other hand, mimosinase expression is high under the condition of nitrogen limitation. Honda and Borthakur (2021) observed positive correlation between mimosine contents of foliage with nitrogen availability. The mimosine molecule has two nitrogen atoms, which constitute 14% of its molecular weight. Therefore, it is expected that nitrogen availability conditions are more conducive for mimsoine synthesis. On the other hand, under the condition of nitrogen limitation, leucaena does not have the nitrogen resource to synthesize mimosine. Under such nitrogen limited conditions, the plant will degrade mimosine to obtain nitrogen from it. Therefore, it is not surprising that mimosinase expression is high under nitrogen deficiency conditions. Mimosine has also been described as a storage molecule, which is synthesized under favorable conditions and degraded under stress conditions such as nitrogen deficiency (Honda and Borthakur, 2021).

Tannin biosynthesis pathway is downstream of phenylpropanoid and flavonoid pathways (Figure 1). These two upstream pathways contribute to flavonol and tannin biosynthesis pathways. Among the five genes for tannin biosynthesis tested in this study, two of them (CHS and F3H) are required for both tannin and flavonol biosynthesis, whereas the remaining three genes (DFR, LAR, and ANR) are more specific for tannin biosynthesis. Overall, tannin synthesis is reduced under salinity conditions. The expression of chalcone synthase appears to be induced by moderate levels of salt. At 50 mM salt concentration, its expression was three times of that without salt. A similar trend was also observed for F3H, which was induced 1.6 fold at 50 mM salt concentration. On the other hand, the expression of DFR, LAR and ANR is inhibited by salt. In *Arabidopsis*, CHS is regulated by the same transcription factor that regulates flavonol synthase (FLS), thus making a stronger connection between transcription of CHS and FLS (Mehrtens et al., 2005). FLS is a key gene for diverting the flavonoid pathway to flavonol synthesis (Figure 1). It has been shown in both *Arabidopsis* and soybean that CHS is involved in conferring resistance to salt stress (Zhu et al., 2021). Therefore, it is not surprising that under salt stress conditions, the leucaena CHS gene is induced. Similarly, the overexpression of F3H in transgenic tobacco confers tolerance to salt stress and infection by *Alternaria solani* (Mahajan and Yadav, 2014). On the other hand, the same salt stress conditions that induced transcription of CHS and F3H inhibited transcription of the DFR gene for tannin biosynthesis in *Arabidopsis* (Espley et al., 2009). In the present study also, the salt stress conditions induced the transcription of CHS and F3H but repressed the transcription of the genes that are directly involved in tannin biosynthesis, namely, DFR, LAR, and ANR. Based on the above discussion, it is apparent that flavonols are mostly responsible for salt stress tolerance in leucaena. The amount of tannin is not directly related to salt tolerance.

Tannin molecules do not contain nitrogen; therefore, nitrogen availability is not expected to be a major factor for tannin biosynthesis. However, nitrogen is a general requirement for plant growth and therefore, nitrogen availability may indirectly influence tannin biosynthesis. Under low nitrogen and drought conditions, plants produce condensed tannins with longer polymer structures (Suseela, 2019). In the present study, tannin production increased two-fold under nitrogen availability condition compared to the nitrogen deficiency condition. This increase in tannin production appeared to be affected by increased expression of the three genes (DFR, LAR, and ANR) that are specific for tannin biosynthesis. On the other hand, the expression of other two genes (CHS and F3H) that are common for both flavonol and tannin biosynthesis pathways, did not show the same trend. Their expression levels were induced under nitrogen deficiency conditions. Flavonols are generally involved in biotic and abiotic stress tolerance (Mahajan and Yadav, 2014). The high expression of these two genes might have contributed to increased flavonol synthesis under nitrogen deficiency stress conditions. Knowledge of environmental conditions that promote or inhibit transcription of the genes for mimosine and tannin biosynthesis may be useful in designing environmental conditions that inhibit transcription of these genes, resulting in reduced levels of these compounds in the leucaena foliage.

## Data Availability Statement

The data presented in the study are deposited in the NCBI repository, accession numbers SRX14364097 and SRX14364098. The sequence data are released. You may find our entire sequence data for common leucaena at: https://www.ncbi.nlm.nih.gov/sra/SRX14364097%5Baccn%5D. You may find our entire sequence data for giant leucaena at: https://www.ncbi.nlm.nih.gov/sra/SRX14364098%5Baccn%5D.

## Author Contributions

AB performed most of the field and laboratory work and wrote the manuscript. AK helped in the bioinformatics analysis of transcriptome data. DB supervised the work and edited the manuscript. All authors contributed to the article and approved the submitted version.

## Conflict of Interest

The authors declare that the research was conducted in the absence of any commercial or financial relationships that could be construed as a potential conflict of interest.

## Publisher’s Note

All claims expressed in this article are solely those of the authors and do not necessarily represent those of their affiliated organizations, or those of the publisher, the editors and the reviewers. Any product that may be evaluated in this article, or claim that may be made by its manufacturer, is not guaranteed or endorsed by the publisher.
