# Supplementary material for: Transcriptional Analyses of Genes Related to Fodder Qualities in Giant Leucaena Under Different Stress Environments
Source: Front Plant Sci. 2022 Jun 16;13:885366. doi: 10.3389/fpls.2022.885366 (PMC9243426; doi:10.3389/fpls.2022.885366)
Supplement: Supplementary file 1 [file Data_Sheet_1.pdf]

Supplementary Figures and Tables

Supplementary Figures

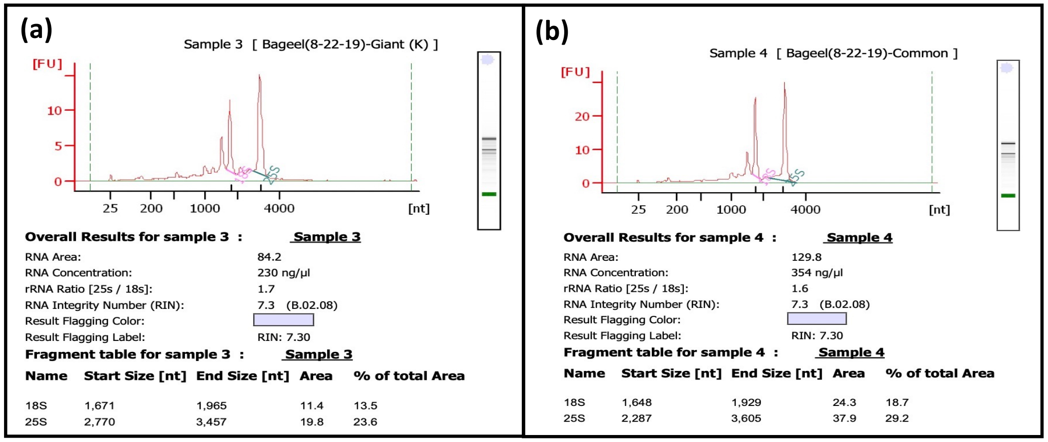

Supplementary Figure 1. RNA Integrity Numbers (RIN) for **(a)** giant and **(b)** common leucaena.

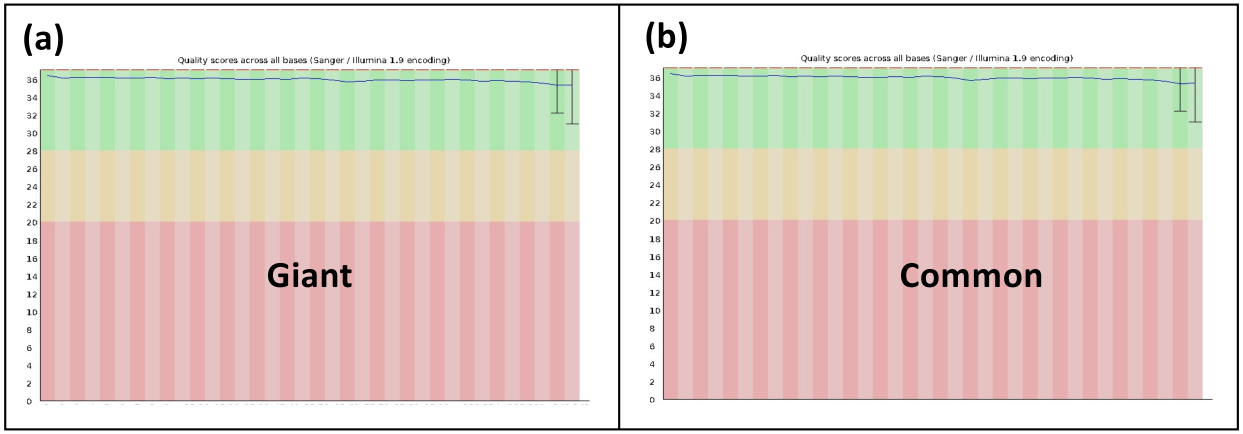

Supplementary Figure 2. FastQC results to show sequence quality Per Base for **(a)** giant and **(b)** common leucaena. The y-axis on the graph shows the quality scores. The higher the score the better the base call. The background of the graph divides the y axis into very good quality calls (green), calls of reasonable quality (orange), and calls of poor quality (red). The central red line is the median value, the blue line represents the mean quality.

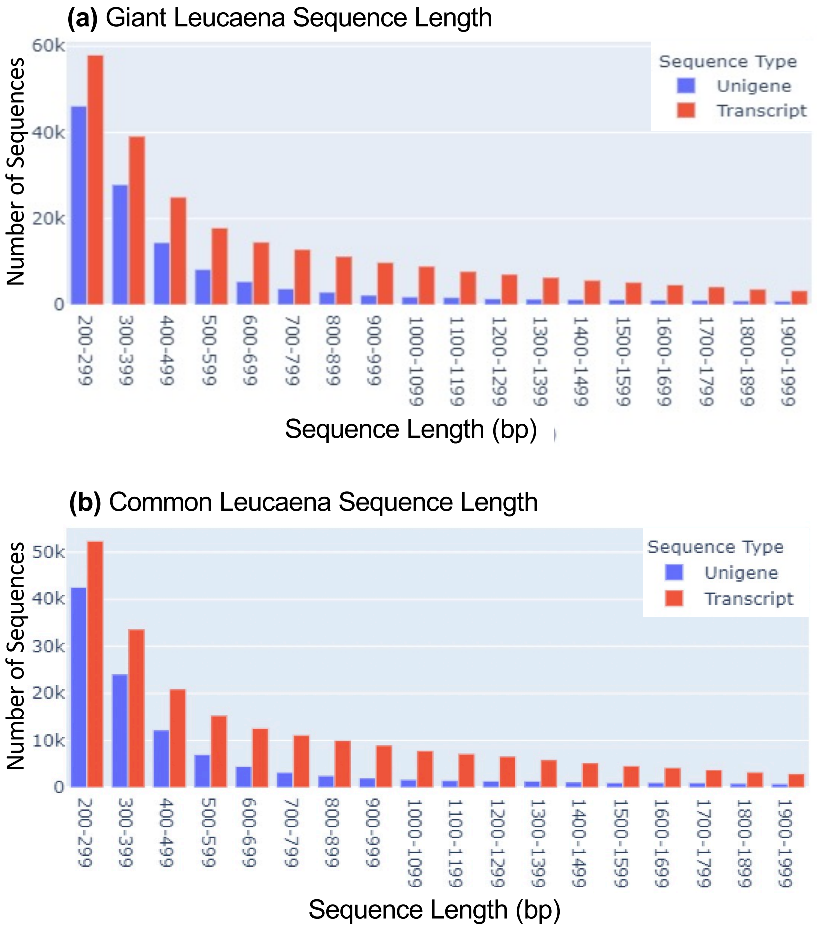

Supplementary Figure 3. The length distribution of **(a)** giant, and **(b)** common leucaena transcripts and their unigene representatives assembled by TRINITY. Sequences are grouped based on their nucleotide length, and the number of sequences in each group is reported.

Supplementary Tables

Supplementary Table 1. Genes showing significant differences in expression between giant and common leucaena (number of transcripts obtained from transcriptome sequencing). The data are based on whole-plant analyses, which include both shoot and root.

Gene	**Giant** (number of transcript)	**Common** (number of transcript)	**Giant** (transcript per million)	**Common** (transcript per million)	**log2 fold change**	**Regulation**	**abs log2 fold change**
mannose/glucose-specific lectin-like protein isoform X2 [*Cinnamomum micranthum* f. *kanehirae*]	11843	105	309.48	9.32	6.41	up	6.41
Abscisic acid receptor PYL6, partial [*Mucuna pruriens*]	24	723	0.84	34.25	-5.31	down	5.31
hypothetical protein PHAVU_010G127400g [*Phaseolus vulgaris*]	33	851	1.40	42.1	-5.09	down	5.09
PLAT domain-containing protein 3-like [*Durio zibethinus*]	18	448	0.40	26.25	-5.03	down	5.03
hypothetical protein TanjilG_03362 [*Lupinus angustifolius*]	1113	30	17.97	0.57	4.8	up	4.8
hypothetical protein DM860_004322 [*Cuscuta australis*]	25	504	1.60	37.78	-4.73	down	4.73
non-specific lipid transfer-like protein [Prosopis juliflora]	11580.3	331.44	1534.36	51.17	4.72	up	4.72
subtilisin-like protease SBT1.7 [*Arachis ipaensis*]	187	3527	2.40	53.09	-4.64	down	4.64
germin-like protein subfamily 1 member 13 isoform X1 [*Abrus precatorius*]	35	607	1.48	29.99	-4.52	down	4.52
4-hydroxyphenylpyruvate dioxygenase [*Cucurbita maxima*]	41	696	0.85	16.84	-4.49	down	4.49
Cysteine-rich RLK (receptor-like protein kinase) 8 [*Dorcoceras hygrometricum*]	458	16	5.17	0.23	4.42	up	4.42
hypothetical protein BVRB_041110, partial [*Beta vulgaris* subsp. *vulgaris*]	41.23	629.99	7.57	83.19	-4.34	down	4.34
protein LURP-one-related 10-like [*Abrus precatorius*]	38	567	2.06	35.84	-4.3	down	4.3
probable xyloglucan endotransglucosylase/hydrolase protein 33 isoform X1 [*Abrus precatorius*]	26	372	0.81	16.34	-4.24	down	4.24
MLP-like protein 31 [*Manihot esculenta*]	52	729	3.22	52.92	-4.21	down	4.21
Auxin-induced protein 10A5 [*Cajanus* cajan]	21	295	1.67	27.45	-4.21	down	4.21

berberine bridge enzyme-like 8 [*Abrus precatorius*]	21	292	0.60	9.73	-4.19	down	4.19
patatin-like protein 3 [*Vigna unguiculata*]	18	235	0.61	9.26	-4.1	down	4.1
hypothetical protein TanjilG_23768 [*Lupinus angustifolius*]	41	530	4	60.94	-4.09	down	4.09
hypothetical protein KK1_042438, partial [*Cajanus cajan*]	20	257	1.57	23.56	-4.08	down	4.08
fasciclin-like arabinogalactan protein 11 [*Glycine max*]	99	1263	2.81	42.11	-4.08	down	4.08
GDSL esterase/lipase isoform A [*Glycine soja*]	19	242	0.74	12	-4.07	down	4.07
unknown [*Glycine max*]	987	45	82.66	4.41	4.05	up	4.05
tryptophan synthase beta chain 1-like [*Vigna unguiculata*]	17	212	0.48	7.07	-4.04	down	4.04
PREDICTED: E3 ubiquitin-protein ligase TRAF7-like [*Nicotiana attenuata*]	99	1223	2.92	41.81	-4.03	down	4.03
Cytochrome P450 [*Macleaya cordata*]	30	356	2.58	31.11	-3.97	down	3.97
abscisic acid receptor PYL4 [*Abrus precatorius*]	191	2258	6.53	90.61	-3.97	down	3.97
hypothetical protein L195_g001899 [*Trifolium pratense*]	40	447	1.49	20.23	-3.88	down	3.88
L-ascorbate oxidase homolog [*Cajanus cajan*]	78	859	1.33	17.58	-3.87	down	3.87
fasciclin-like arabinogalactan protein 12 [*Abrus precatorius*]	18	195	1.74	11.1	-3.83	down	3.83
hypothetical protein GLYMA_13G013300 [*Glycine max*]	26.51	290.77	6.22	23.51	-3.83	down	3.83
glucan endo-1,3-beta-glucosidase [*Medicago truncatula*]	26	280	1.06	12.5	-3.83	down	3.83
PREDICTED: fasciclin-like arabinogalactan protein 12 [*Vigna angularis*]	48	513	2.04	28.3	-3.82	down	3.82
UDP-glycosyltransferase 74E1-like [*Quercus suber*]	963	54	35.69	2.49	3.75	up	3.75
Putative xyloglucan endotransglucosylase/hydrolase protein 23 [*Glycine soja*]	89	899.3	13.64	114.78	-3.74	down	3.74
putative xyloglucan endotransglucosylase/hydrolase protein 23 [*Mucuna pruriens*]	53	534	10.59	124.06	-3.74	down	3.74
allene oxide synthase [*Camellia sinensis*]	74	742	1.59	18.9	-3.73	down	3.73
hypothetical protein CDL15_Pgr016053 [*Punica granatum*]	55	544	0.93	10.76	-3.71	down	3.71

heavy metal-associated isoprenylated plant protein 23 isoform X1 [*Abrus precatorius*]	38	356	3.84	30.48	-3.63	down	3.63
UPF0481 protein At3g47200-like [*Abrus precatorius*]	673	41	16	5.34	3.63	up	3.63
hypothetical protein COLO4_01417 [Corchorus olitorius]	19	178	1.71	19.91	-3.62	down	3.62
terpene synthase 10-like [*Abrus precatorius*]	26	243	0.48	5.27	-3.62	down	3.62
hypothetical protein Ahy_B01g053765 isoform B [*Arachis hypogaea*]	34	317	5.83	70.37	-3.62	down	3.62
caffeic acid 3-O-methyltransferase-like [*Cajanus cajan*]	103	936	4.86	51.71	-3.59	down	3.59
thaumatin-like protein 1 [*Abrus precatorius*]	72	642	2.56	26.91	-3.56	down	3.56
peroxidase 21 [*Cajanus cajan*]	137.14	1213.83	4.64	47.08	-3.55	down	3.55
allene oxide synthase 3-like [*Abrus precatorius*]	153	1352	2.99	30.94	-3.55	down	3.55
Exocyst complex component SEC10 [*Zea mays*]	24.18	212.46	2.88	30.93	-3.54	down	3.54
bidirectional sugar transporter SWEET3-like [*Abrus precatorius*]	44	387	1.9	20.19	-3.54	down	3.54
9-lipoxygenase, partial [*Nicotiana benthamiana*]	245	16.25	11.07	0.86	3.52	up	3.52

Supplementary Table 2. Number of unigenes associated with mimosine and tannin metabolism. The data are based on transcriptome sequencing of whole-plant, which includes both shoot and root.

Genes of Interest	Number of Associated Unigenes	
	Giant	Common
Mimosine Synthase	44	61
Mimosinase	54	35
Chalcone Synthase	29	18
Flavanone 3ß-Hydroxylase	75	84
Leucoanthocyanidin Reductase	11	7
Dihydroflavanol Reductase	43	49
Anthocyanidin Reductase	33	41
